# Supplementary material for: Timeline of changes in spike conformational dynamics in emergent SARS-CoV-2 variants reveal progressive stabilization of trimer stalk with altered NTD dynamics
Source: eLife. 2023 Mar 17;12:e82584. doi: 10.7554/eLife.82584 (PMC10049203; doi:10.7554/eLife.82584)
Supplement: Supplementary file 1. — N-linked glycans were identified by mass spectrometry. The number of glycans identified at each site and an example glycan are reported. [file elife-82584-supp1.docx]

Table S1.

|  | **Wildtype** | | **D614G** | | **Delta** | | **Omicron** | |
| --- | --- | --- | --- | --- | --- | --- | --- | --- |
|  | **Number of unique glycans detected** | **Example Glycan** | **Number of unique glycans detected** | **Example Glycan** | **Number of unique glycans detected** | **Example Glycan** | **Number of unique glycans detected** | **Example Glycan** |
| **N17** | 2 | HexNAc(4)Hex(6)Fuc(1)NeuAc(1) | 3 | HexNAc(4)Hex(6)NeuAc(1) | 0 | N/A | 2 | HexNAc(5)Hex(4) |
| **N61** | 40 | HexNAc(4)Hex(5)Fuc(1)NeuAc(1) | 25 | HexNAc(4)Hex(5)NeuAc(2)Na(2) | 30 | HexNAc(5)Hex(6)NeuAc(1) | 12 | HexNAc(6)Hex(3)Fuc(1)NeuAc(2) |
| **N74** | 43 | HexNAc(6)Hex(3)Fuc(1)NeuAc(1) | 41 | HexNAc(4)Hex(5)Fuc(1)NeuAc(2) | 40 | HexNAc(6)Hex(3)Fuc(1)NeuAc(1) | 9 | HexNAc(4)Hex(5)Fuc(1) |
| **N122** | 42 | HexNAc(4)Hex(5)Fuc(1)NeuAc(2)Na(1) | 41 | HexNAc(3)Hex(6)Fuc(1)NeuAc(1) | 36 | HexNAc(3)Hex(4)NeuAc(1) | 39 | HexNAc(4)Hex(5)Fuc(1)NeuAc(2)Na(2) |
| **N149** | 2 | HexNAc(2)Hex(5) | 2 | HexNAc(4)Hex(5)Fuc(1)NeuAc(2)Na(1) | 23 | HexNAc(3)Hex(6)Fuc(1)NeuAc(1) | 0 | N/A |
| **N165** | 6 | HexNAc(4)Hex(4)NeuAc(1)Na(1) | 7 | HexNAc(4)Hex(4)NeuAc(1)Na(1) | 25 | HexNAc(4)Hex(6)Fuc(1)NeuAc(1)Na(1) | 8 | HexNAc(4)Hex(4)NeuAc(1)Na(1) |
| **N234** | 33 | HexNAc(4)Hex(6)Fuc(1)NeuAc(1)Na(1) | 29 | HexNAc(4)Hex(6)Fuc(1)NeuAc(1) | 27 | HexNAc(4)Hex(5)NeuAc(2)Na(2) | 18 | HexNAc(4)Hex(6)Fuc(1)NeuAc(1) |
| **N282** | 7 | HexNAc(6)Hex(3)Fuc(1)NeuAc(2) | 17 | HexNAc(4)Hex(5)Fuc(1)NeuAc(1)Na(1) | 11 | HexNAc(4)Hex(5)NeuAc(2)Na(2) | 10 | HexNAc(4)Hex(5)Fuc(1)NeuAc(1)Na(1) |
| **N331** | 3 | HexNAc(3)Hex(6)Fuc(1)NeuAc(1) | 3 | HexNAc(3)Hex(4)NeuAc(1) | 2 | HexNAc(4)Hex(5)Fuc(1)NeuAc(1)Na(1) | 0 | N/A |
| **N343** | 4 | HexNAc(4)Hex(5)NeuAc(2)Na(1) | 6 | HexNAc(4)Hex(5)NeuAc(2)Na(1) | 4 | HexNAc(4)Hex(5)NeuAc(2)Na(1) | 7 | HexNAc(6)Hex(3)Fuc(1)NeuAc(2) |
| **N603** | 12 | HexNAc(4)Hex(5)Fuc(1)NeuAc(1)Na(1) | 7 | HexNAc(4)Hex(5)Fuc(1)NeuAc(2)Na(1) | 16 | HexNAc(6)Hex(3)Fuc(1)NeuAc(1) | 3 | HexNAc(5)Hex(5)NeuAc(1) |
| **N616** | 13 | HexNAc(4)Hex(5)Fuc(1)NeuAc(2) | 9 | HexNAc(4)Hex(5)NeuAc(1)Na(1) | 18 | HexNAc(4)Hex(5)Fuc(1)NeuAc(2) | 2 | HexNAc(2)Hex(9) |
| **N657** | 0 | N/A | 0 | N/A | 5 | HexNAc(6)Hex(3)Fuc(1)NeuAc(2) | 0 | N/A |
| **N709** | 5 | HexNAc(4)Hex(6)Fuc(1)NeuAc(1)Na(1) | 1 | HexNAc(5)Hex(6)NeuAc(1) | 17 | HexNAc(4)Hex(5)Fuc(1)NeuAc(2) | 3 | HexNAc(4)Hex(5)NeuAc(1) |
| **N717** | 14 | HexNAc(4)Hex(5)Fuc(1)NeuAc(2) | 7 | HexNAc(4)Hex(5)Fuc(1)NeuAc(1)Na(1) | 25 | HexNAc(6)Hex(3)Fuc(1)NeuAc(2) | 4 | HexNAc(4)Hex(5)Fuc(1)NeuAc(2)Na(2) |
| **N801** | 44 | HexNAc(4)Hex(5)Fuc(1)NeuAc(1) | 46 | HexNAc(4)Hex(5)Fuc(1)NeuAc(1) | 42 | HexNAc(6)Hex(3)Fuc(1)NeuAc(1) | 40 | HexNAc(3)Hex(6)Fuc(1)NeuAc(1) |
| **N1074** | 0 | N/A | 7 | HexNAc(4)Hex(5)NeuAc(2)Na(1) | 1 | HexNAc(4)Hex(5)NeuAc(1) | 7 | HexNAc(4)Hex(5)Fuc(1)NeuAc(1) |
| **N1098** | 50 | HexNAc(4)Hex(5)Fuc(1)NeuAc(2)Na(2) | 52 | HexNAc(4)Hex(5)Fuc(1)NeuAc(1) | 52 | HexNAc(4)Hex(5)Fuc(1)NeuAc(2)Na(2) | 49 | HexNAc(4)Hex(5)Fuc(1)NeuAc(2) |
| **N1134** | 5 | HexNAc(4)Hex(5)NeuAc(2)Na(1) | 12 | HexNAc(4)Hex(6)Fuc(1)NeuAc(1) | 21 | HexNAc(6)Hex(3)Fuc(1)NeuAc(1) | 5 | HexNAc(3)Hex(6)Fuc(1)NeuAc(1) |
| **N1158** | 12 | HexNAc(4)Hex(5)Fuc(1)NeuAc(1) | 6 | HexNAc(4)Hex(5)NeuAc(2)Na(1) | 7 | HexNAc(4)Hex(4)NeuAc(1) | 9 | HexNAc(4)Hex(5)Fuc(1)NeuAc(1) |
| **N1173** | 11 | HexNAc(6)Hex(3)Fuc(1)NeuAc(2) | 6 | HexNAc(6)Hex(4)NeuAc(1) | 7 | HexNAc(6)Hex(3)Fuc(1)NeuAc(2) | 9 | HexNAc(6)Hex(3)Fuc(1)NeuAc(2) |
| **N1194** | 23 | HexNAc(4)Hex(5)NeuAc(2)Na(2) | 30 | HexNAc(4)Hex(5)Fuc(1)NeuAc(1) | 27 | HexNAc(4)Hex(5)Fuc(1)NeuAc(1) | 14 | HexNAc(4)Hex(5)Fuc(1)NeuAc(1) |
