# Supplementary material for: Timeline of changes in spike conformational dynamics in emergent SARS-CoV-2 variants reveal progressive stabilization of trimer stalk with altered NTD dynamics
Source: eLife. 2023 Mar 17;12:e82584. doi: 10.7554/eLife.82584 (PMC10049203; doi:10.7554/eLife.82584)
Supplement: Supplementary file 2. — Differences between variants and D614G S for mutated peptides reported in Da for Dex = 1, 2, 10, and 30 min exchange. Mutations sites are bolded and underlined. [file elife-82584-supp2.docx]

**Table S2.**

| **Peptide** | **Sequence** | **Variant** | **Δ Da 1 min** | **Δ Da 2 min** | **Δ Da 10 min** | **Δ Da 30 min** |
| --- | --- | --- | --- | --- | --- | --- |
| 92-103 | FAS**I**EKSNIIRG | Omicron | -0.2 ± 0.1 | 0.2 ± 0.1 | 0.9 ± 0.1 | 0.9 ± 0.1 |
| 401-420 | VIRGDEVRQIAPGQTG**N**IAD | Omicron | 0.6 ± 0.3 | 0.7 ± 0.3 | 0.5 ± 0.3 | 0.3 ± 0.3 |
| 401-421 | VIRGDEVRQIAPGQTG**N**IADY | Omicron | 0.5 ± 0.3 | 0.9 ± 0.3 | 0.4 ± 0.3 | 0.3 ± 0.3 |
| 407-420 | VRQIAPGQTG**N**IAD | Omicron | 0.6 ± 0.2 | 0.7 ± 0.2 | 0.5 ± 0.2 | 0.5 ± 0.2 |
| 407-421 | VRQIAPGQTG**N**IADY | Omicron | 0.5 ± 0.3 | 0.8 ± 0.3 | 0.6 ± 0.3 | 0.3 ± 0.3 |
| 407-422 | VRQIAPGQTG**N**IADYN | Omicron | -0.4 ± 0.1 | -0.3 ± 0.1 | -0.1 ± 0.1 | 0.1 ± 0.1 |
| 442-452 | DSKV**S**GNYNYL | Omicron | 0.3 ± 0.2 | 0.5 ± 0.2 | 0.1 ± 0.2 | -0.2 ± 0.2 |
| 444-452 | KV**S**GNYNYL | Omicron | -1.5 ± 0.2 | -1.3 ± 0.2 | -1.2 ± 0.2 | -1.1 ± 0.2 |
| 542-552 | NFNGL**K**GTGVL | Omicron | -0.2 ± 0.1 | -0.2 ± 0.1 | -0.5 ± 0.1 | -0.5 ± 0.1 |
| 544-552 | NGL**K**GTGVL | Omicron | 0.1 ± 0.1 | 0.0 ± 0.1 | -0.2 ± 0.1 | -0.2 ± 0.1 |
| 569-582 | I**D**DTTDAVRDPQTL | Alpha | -0.2 ± 0.3 | -0.3 ± 0.3 | -0.4 ± 0.3 | -0.5 ± 0.3 |
| 761-768 | **K**RALTGIA | Omicron | -0.2 ± 0.2 | -0.1 ± 0.2 | -0.2 ± 0.2 | -0.3 ± 0.2 |
| 780-793 | AQVKQIYKTPPIK**Y** | Omicron | -0.2 ± 0.3 | -0.1 ± 0.3 | -0.1 ± 0.3 | -0.2 ± 0.3 |
| 943-958 | GKLQ**N**VVNQNAQALNT | Delta | 1.1 ± 0.4 | 0.3 ± 0.4 | 0.2 ± 0.4 | -0.2 ± 0.4 |
| 943-958 | GKLQ**N**VVNQNAQALNT | Omicron | -1.9 ± 0.3 | -1.3 ± 0.3 | -1.3 ± 0.3 | -1.7 ± 0.3 |
| 959-967 | LVKQLSS**K**F | Omicron | 0.8 ± 0.1 | 0.8 ± 0.1 | 0.2 ± 0.1 | 0.1 ± 0.1 |
| 959-974 | LVKQLSS**K**FGAISSVL | Omicron | 2.1 ± 0.3 | 1.9 ± 0.3 | 1.9 ± 0.3 | 1.7 ± 0.3 |
| 977-987 | IL**A**RLDPPEAE | Alpha | -0.8 ± 0.2 | -0.7 ± 0.2 | -0.4 ± 0.2 | -0.1 ± 0.2 |
| 977-987 | I**F**SRLDPPEAE | Omicron | 0.7 ± 0.2 | 0.5 ± 0.2 | -0.2 ± 0.2 | -0.4 ± 0.2 |
| 979-987 | **A**RLDPPEAE | Alpha | -0.4 ± 0.2 | -0.4 ± 0.2 | -0.9 ± 0.2 | -1.0 ± 0.2 |
| 979-989 | **A**RLDPPEAEVQ | Alpha | -0.6 ± 0.2 | -0.4 ± 0.2 | -0.5 ± 0.2 | -0.4 ± 0.2 |
