# Supplementary material for: Timeline of changes in spike conformational dynamics in emergent SARS-CoV-2 variants reveal progressive stabilization of trimer stalk with altered NTD dynamics
Source: eLife. 2023 Mar 17;12:e82584. doi: 10.7554/eLife.82584 (PMC10049203; doi:10.7554/eLife.82584)
Supplement: Supplementary file 3. — Experimental conditions and coverage for HDXMS analysis of WT and variant S. [file elife-82584-supp3.docx]

**Table S3**

| **Spike Dataset** | **WT S 6P vs WT S 2P** | **WT S-3h 37C vs WT S-no incubation** | **D614G S-3h 37C vs D614G S-no incubation** | **D614G S vs WT S** | **Alpha S vs D614G S** | **Delta S vs Alpha S** | **Omicron BA.1 S vs Delta S** |
| --- | --- | --- | --- | --- | --- | --- | --- |
| **HDX reaction details** | Labeling buffer: Phosphate Buffered Saline (PBS) pH 7.4 prepared in 99.9% D_2_O to a final concentration of 94.9% D_2_O  Quench buffer: 1.5 M Guandinium hydrochloride, 0.25 M TCEP  HDXMS reaction: 57 μL Labeling buffer added to 3 μL S sample (12 μM) for deuterium exchange at 20°C and quenched with 60 μL of quench buffer by bringing reaction to pH 2.5 at 0°C. | | | | | | |
| **Spike incubation** | No incubation | 1. WT- 3h, 37°C 2. WT- no incubation | 1. D614G- 3h at 37°C 2. D614G- no incubation | 3h at 37°C | | | |
| **HDXMS un-deuterated controls** | 57 μL of PBS pH 7.4 was added to 3 μL S sample (12 μM) and 60 μL of quench buffer was added by bringing reaction to pH 2.5 at 0°C. | | | | | | |
| **HDXMS time course**  **(min)** | 1, 2, 10 | | | 1, 2, 10, 30 | | | |
| **Back exchange** | 20% | | | | | | |
| **Number of peptides** | 160 | 127 | 133 | 120 | 127 | 123 | 95 |
| **Sequence coverage** | 53.1% | 48.4% | 48.1% | 45.8% | 45.9% | 47.0% | 36.4% |
| **Peptide redundancy** | 2.29 | 2.09 | 2.19 | 2.09 | 2.18 | 2.05 | 1.99 |
| **Replicates** | 3 technical replicates were acquired. During HDXMS time course, samples, excluding WT, were stored at 0°C and each replicate run was staggered to eliminate variability arising from storage time. | | | | | | |
| **Significance difference threshold** | 0.5 Da as represented in deuterium exchange difference plots.  Hybrid significance testing was utilized to determine the significance threshold for peptides corresponding to a p-value < 0.01 and represented as a Volcano plot (Lau. et al 2021). | | | | | | |
